# Biocontrol of *Fusarium graminearum*, a Causal Agent of Fusarium Head Blight of Wheat, and Deoxynivalenol Accumulation: From *In Vitro* to *In Planta*

**DOI:** 10.3390/toxins14050299

**Published:** 2022-04-22

**Authors:** Asmaa Abbas, Tapani Yli-Mattila

**Affiliations:** Department of Life Technologies, Faculty of Technology, University of Turku, FI-20014 Turku, Finland; asmaa.abbas@utu.fi

**Keywords:** *in vitro* bioassay, *Zanthoxylum bungeanum*, wheat, *Fusarium graminearum*, mycotoxin, biocontrol

## Abstract

Crop diseases caused by *Fusarium* *graminearum* threaten crop production in both commercial and smallholder farming. *F. graminearum* produces deoxynivalenol mycotoxin, which is stable during food and feed processing. Therefore, the best way to prevent the sporulation of pathogens is to develop new prevention strategies. Plant-based pesticides, i.e., natural fungicides, have recently gained interest in crop protection as alternatives to synthetic fungicides. Herein we show that treatment with the methanolic extract of medicinal plant *Zanthoxylum bungeanum* (M20 extract), decreased *F. graminearum* growth and abrogated DON production. The *F. graminearum* DNA levels were monitored by a quantitative TaqMan real-time PCR, while DON accumulation was assessed by HPLC quantification. This M20 extract was mainly composed of four flavonoids: quercetin, epicatechin, kaempferol-3-O-rhamnoside, and hyperoside. The *in vitro* bioassay, which measured the percent inhibition of fungal growth, showed that co-inoculation of four *F. graminearum* strains with the M20 extract inhibited the fungal growth up to 48.5%. After biocontrol treatments, *F. graminearum* DNA level was reduced up to 85.5% compared to that of wheat heads, which received *F. graminearum* mixture only. Moreover, DON production was decreased in wheat heads by 73% after biocontrol treatment; meanwhile in wheat heads inoculated with *F. graminearum* conidia, an average of 2.263 ± 0.8 mg/kg DON was detected. Overall, this study is a successful case from *in vitro* research to *in planta*, giving useful information for wheat protection against *F. graminearum* responsible for Fusarium Head Blight and DON accumulation in grains. Further studies are needed to study the mechanism by which M20 extract inhibited the DON production and what changes happened to the DON biosynthetic pathway genes.

## 1. Introduction

Many *Fusarium* species are considered phytopathogenic fungi, which mainly lead to Fusarium head blight (FHB) in small-grain cereals, such as wheat, barley, triticale, and oats [1]. Fusarium head blight (FHB), is a global problem because it has great economic burden on the cereal industry due to its significant reductions in grain yield and quality [2]. Upon infection, several *Fusarium* species produce aggressive secondary metabolites, which lead to crop contamination such as, deoxynivalenol (DON), nivalenol (NIV), T2 toxin, fumonisin (FUM), and mycoestrogen zearalenone (ZEN) [3]. Consumption of trichothecenes is toxic to humans and animals, they cause intestinal irritation, feed refusal in livestock, vomiting, skin dermatitis, immunosuppression, anorexia, and growth retardation [4,5]. As a result, the European commission (EC) has set maximum limits to control the use of food and feedstuff contaminated with DON and ZEN mycotoxins. For unprocessed wheat and barley grains, the limits are 1.25 mg/kg DON and 0.1 mg/kg ZEN, while in oat the DON limit is 1.75 mg/kg.

Worldwide, the most prevalent FHB-causing species in wheat belong to *Fusarium graminearum* species complex [6]. FHB has infected many temperate areas, such as East Asia, North America, and Europe. In the past decade, mycotoxin contamination has prompted basic research on the fungal causal agent. *Fusarium graminearum* has become one of the most studied fungal phytopathogens and is considered the fourth most aggressive plant-pathogenic fungus in the world [7,8]. Considering the negative effects on farmers and buyers, the financial loss caused by FHB and mycotoxin contamination in wheat and barley only in the USA between 2016 and 2017 is USD 1.47 billion [9].

Chemical fungicides have been used as a control strategy to stop FHB incidence and DON accumulation over the last four decades. Wheat farmers have applied benzimidazole fungicides, mostly carbendazim in the field [10]. Nevertheless, these chemical treatments are prohibited from two to three weeks before harvesting, despite *F. graminearum* infections and DON contaminations might occur during this period [3]. In addition, resistant varieties and crop rotation are some of the control strategies used [11]. These control strategies have efficiently decreased due to resistance development, promoting *Fusarium* growth or DON production under favorable environmental conditions [6,12]. Use of carbendazim has been prohibited in the EU and the United States [13]. Therefore, other effective approaches are urgently needed to manage FHB in order to satisfy consumer needs. Biological control methods have been recently studied a lot to prevent *F. graminearum* growth or DON secretion. For example, *Clonostachys rosea* fungi reduced the survival of *F. graminearum* on wheat and maize [14] and DON accumulation up to 33% in the infected grains [15]. An antibiotic called validamycin, produced by *Streptomyces hygroscopicus var. limoneus* decreased the activity of trehalase and the production of glucose and pyruvate, which are the precursors of DON pathway and hence inhibited DON synthesis [16]. Some microorganisms also showed effectiveness in reducing disease severity such as, *Streptomyces* [17], *Bacillus subtilis* RC 218 and *Brevibacillus* sp. RC 263 [18], and *Paenibacillus polymyxa* strains [19].

In addition, the use of organic extracts from medicinal plants has attracted attention of researchers after noticing the violence effects of synthetic pesticides on the general health and the environment [20]. These plant extracts can control crop diseases during post-harvest storage and treatment of many diseases. This is due to their beneficial constituents such as phenols, polyphenols, flavonoids, tannins, and alkaloids [21]. Furthermore, the usage of plant extracts against pathogens inhibits the resistance development because they contain antimicrobial compounds and their synergisms [22]. After applying the plant extracts in the post-harvest stage, they biodegraded quickly because natural products are unstable at high temperatures contrary to chemical fungicides which can exist in the environment for a long time [23]. The antifungal activity of curcuma longa extract against *F. graminearum* has been studied and showed that it possesses high antifungal activity with IC50 value 0.1088 mg/mL [24]. Mustard-based botanicals were efficient to control *F. graminearum* due to the presence of phenolic acids in their contents [1].

*Zanthoxylum bungeanum* is a medicinal plant, distributed in China and some Southern Asian countries [25]. It is commonly used for the treatment of abdominal pain, toothache, dyspepsia, vomiting, diarrhea, ascariasis, and eczema [26]. To understand its mode of action, researchers identified its chemical constituents and found that it has 140 chemical compounds including alkaloids, terpenoids, flavonoids, and free fatty acids, and a small amount of inorganic elements [27]. Essential oils from *Z. bungeanum* (EOZB) had a broad spectrum of activity against pathogenic fungi measured by the mycelial growth inhibition method. EOZB inhibited the growth of eleven fungal species with low IC50 values including *Fusarium oxysporum* and *Fusarium sulphureum* [28,29]. To monitor the crop grains, an applicable DNA-based quantification method on the species level is required. At present, DNA extraction is cheap, fast, and reproducible from a wide range of food and grain samples. Together with the rapid, accurate, and highly sensitive quantitative real-time PCR (qPCR) technique, this is a preferential approach to accurately quantify pathogens and antagonists biomass [30].

The objective of this study is to investigate the effect of methanolic extract of *Zanthoxylum bungeanum* plant on *F. graminearum* infection and deoxynivalenol accumulation in wheat. As a first step, the effect of M20 extract was tested using a mycelium growth *in vitro* bioassay on four *F. graminearum* isolates selected from our previous study [31]. Second, the efficacy of M20 extract from *Zanthoxylum bungeanum* [32] was investigated under field conditions in wheat using point inoculation method with either *F. graminearum* mixture or mixture of *F. graminearum* followed by M20 extract in order to evaluate the effect of main flavonoid compounds of M20 extract on *F. graminearum* growth during mid-flowering. Finally, the amount of DON present in the wheat plants was quantified.

## 2. Results

### 2.1. In Vitro Bioassay—Effect of M20 Extract on Mycelium Growth

The study included four different groups: control group received DMSO, group I received mixture of four *F. graminearum* (Fg) isolates, group II received Fg isolates and 100 µg/mL M20 extract, and group III received 100 µg/mL M20 extract only. Quantity of *F. graminearum* DNA was determined by qPCR based on the *TMFg12* gene. The amount of DON produced was monitored by HPLC. In addition, Pearson’s correlation coefficient between *Fusarium* DNA and mycotoxin level was calculated in the four studied groups.

**Figure 1:**
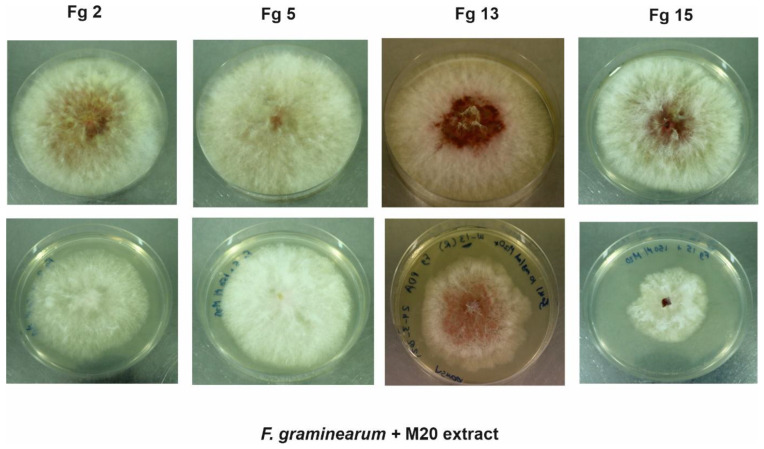
The first row of the PDA plates shows the control plates (no M20 extract) for the four strains, the second row of the PDA plates shows Fg strains incubated with 150 µg/mL of M20 extract in the dark for five days. Fg: *Fusarium graminearum*. Fg 2 and Fg 5 are Finnish strains, Fg 13 and Fg 15 are Russian strains.

**Figure 2:**
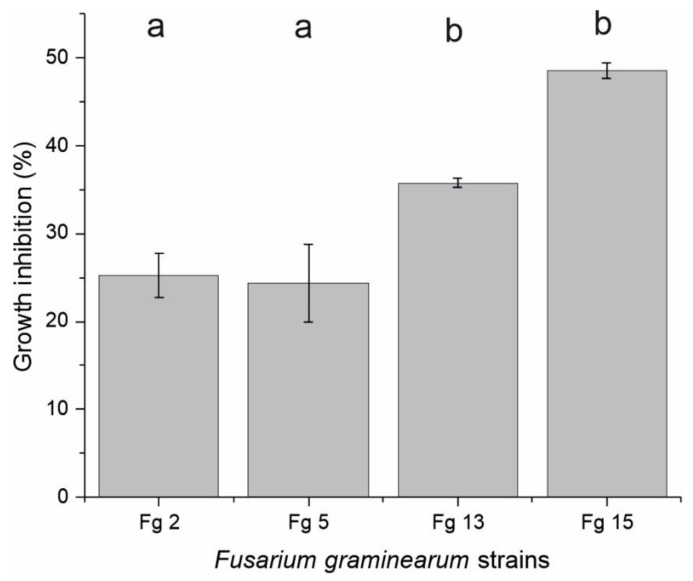
Percent of growth inhibition of four different *F. graminearum* isolates after 5 days incubation with 150 µg/mL of M20 extract on PDA plates. Control plates inoculated with DMSO instead of the treated extract. All experiments were performed in triplicates. Data are presented as mean ± standard deviation (S.D). Columns indicated with the same letters are not significantly different (*p* > 0.05) and columns indicated with different letters are significantly different (*p* < 0.05) according to Tukey’s test.

In the laboratory, the growth of the phytopathogen *F. graminearum* was monitored in the presence and absence of 150 µg/mL of M20 extract (Figure 1 and Figure 2). The extract showed consistent antifungal activities against the Finnish and Russian *F. graminearum* strains. Hyphal growth of the Finnish strains (Fg 2 and Fg 5) was inhibited at a range between 24–25%. However, the Russian strains Fg 15 showed the highest (48.5%) reduction in hyphal growth, followed by the other Russian strain Fg 13 which showed 35.5% inhibition percentage.

### 2.2. Quantification of F. graminearum DNA in Wheat Using qPCR

**Figure 3:**
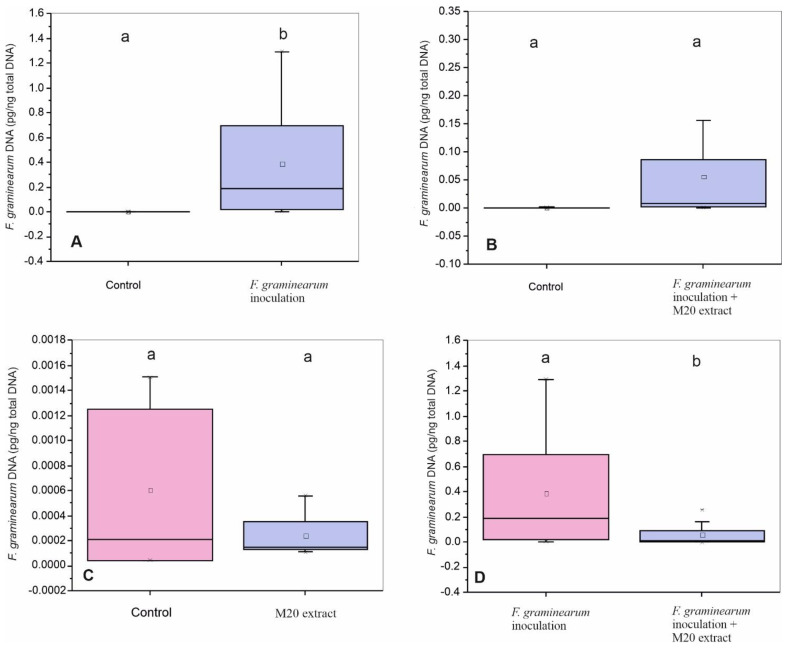
Suppression effect of M20 extract on *F. graminearum* DNA level (pg/ng total DNA) in wheat grains. Box-plots with the same letters on top of the graph are not significantly different (*p* > 0.05). Box-plots with different letters on top of the graph are significantly different (*p* < 0.05) according to Tukey’s test. (**A**) *F. graminearum* DNA level (pg/ng total DNA) in control and group I. (**B**) *F. graminearum* DNA level (pg/ng total DNA) in control and group II. (**C**) *F. graminearum* DNA level (pg/ng total DNA) in control and group III. (**D**) *F. graminearum* DNA level (pg/ng total DNA) in group I and group II.

All samples (except the negative control samples) showed a positive signal above the detection limit within an amplification range between 23 and 37 cycles. QPCR amplifications of control samples (no-M20 extract or fungal treatment) showed only traces of *F. graminearum* DNA levels, approving that the natural infection level was low. Point inoculation method that we used in the field succeeded in growing *F. graminearum* fungi in treatment I as it is clear from Figure 3A, that there is a significance between the fungal DNA amount between control (DMSO group) and treatment I (Fg group). Abundance of *F. graminearum* in treatments II and III were not significantly different from the control in Figure 3B,C, respectively. However, after inoculating Fg wheat samples with M20 extract (treatment II), *Fusarium* abundance was reduced up to 85.5% compared to treatment I and significantly reduced *F. graminearum* disease severity in treatment II (Figure 3D).

### 2.3. M20 Extract Inhibited DON Production by F. graminearum

**Figure 4:**
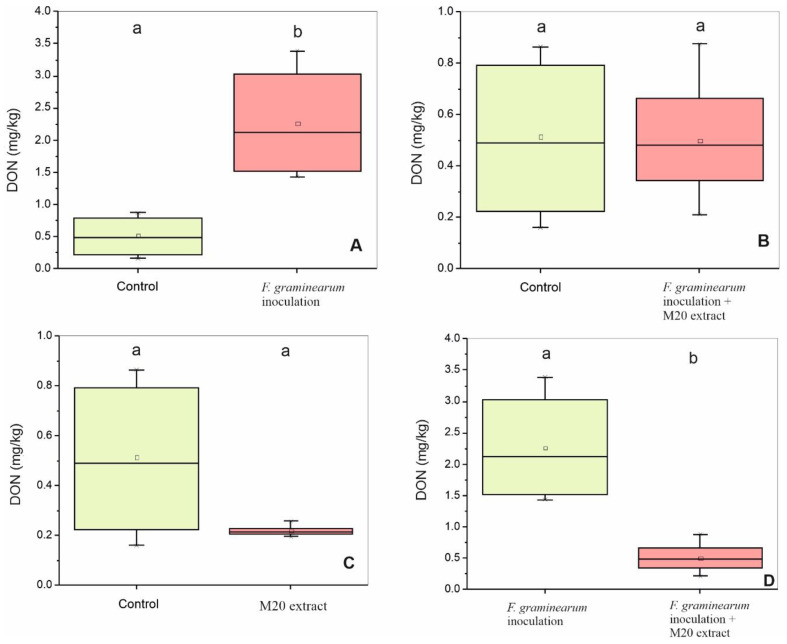
Suppression effect of M20 extract on DON level in wheat. ANOVA analysis was performed and Tukey test used to identify treatments significantly different from pathogen group I. Box-plots with the same letters on top of the graph are not significantly different (*p* > 0.05); however, different letters indicate statistically significant differences (*p* < 0.05). (**A**) DON level (mg/kg) in control and group I. (**B**) DON level (mg/kg) in control and group II. (**C**) DON level (mg/kg) in control and group III. (**D**) DON level (mg/kg) in group I and group II.

To verify the ability of M20 extract to reduce the mycotoxin accumulation, the amount of DON extracted from grinded wheat samples was measured with HPLC and was expressed as mg/kg wheat. Heads inoculated with the pathogens only (treatment I) revealed the highest amount of DON (2.263 ± 0.8 mg/kg) and it was significantly higher than the control group which received DMSO only, and led to an average DON content of 0.515 ± 0.3 mg/kg (Figure 4A). Furthermore, there were no significant changes between control and treatments II (received Fg conidia mix and M20 extract) and III (received M20 extract only) (Figure 4B,C) respectively. Application of M20 extract significantly reduced the DON content in treatment II by 73% compared to treatment I (Figure 4D). In general, lower DON incidence was observed in treatment III (which was treated with M20 extract only) in comparison with the other groups.

### 2.4. Correlation between Fungal DNA and DON Content

**Figure 5:**
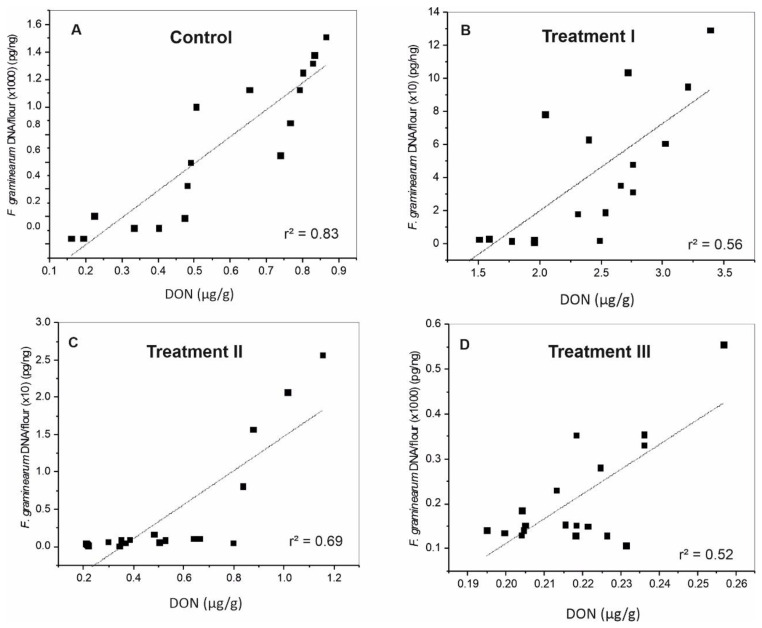
Scatter diagrams show the correlation between *F. graminearum* DNA and DON levels in wheat grains in the four studied treatments. *F. graminearum* DNA was multiplied by 1000 or 10 in order to be in the normal range with the toxin data. (**A**) Control (received DMSO) (**B**) Group I (received Fg mixture) (**C**) Group II (received Fg mixture and 100 µg/mL M20 extract) (**D**) Group III (received 100 µg/mL M20 extract). r^2^: coefficient of determination.

Coefficient of determination (r^2^) between *F. graminearum* DNA and DON level was calculated using the data from all inoculated groups. In general, the highest value was observed in the controls (without Fg or M20 extract) (Figure 5A). Relatively high coefficient of determination, 0.56, 0.69, and 0.52 was also found for treatments I, II, and III respectively (Figure 5B–D).

## 3. Discussion

Our study contributes to interpreting the potential use of methanolic extract from *Zanthoxylum bungeanum* plant against *F. graminearum* infection and deoxynivalenol accumulation in wheat under controlled environment and under field conditions. The negative economic impact of *Fusarium* infection in wheat increases by the combination of FHB and mycotoxin incidence in the grains harvested from infected fields. For that reason, the biocontrol of *F. graminearum* with natural fungicides is a highly desirable alternative way instead of using synthetic fungicides. Chemical constituents of the natural plants such as, phenolic compounds and essential oils are a promising replacement for synthetic fungicides because plants produce many different compounds either as part of their development or in reaction to stress or pathogens [33,34]. Essential oils of *Z. bungeanum* have been known to have strong anti-bacterial and anti-fungal effects. They have strong inhibitory effect on *Bacillus*, *Saccharomyces cerevisiae*, and *Aspergillus* species [35]. Our previous study by Abbas et al., [32] has fractionated the crude methanolic extract of *Z. bungeanum* to twelve sub-fractions and results showed that M20 extract had the highest phenolic/flavonoid contents and antioxidant activities. This extract contained four flavonoids, quercetin, epicatechin, kaempferol-3-O-rhamnoside, and hyperoside as identified by mass spectrometry analyses. Therefore, in this study, we investigated the effect of these flavonoids found in M20 extract on *F. graminearum* development. Phenolic and flavonoid compounds are known for their antioxidant properties as, they can bind free radicals and decrease the risk of chronic diseases. The most common phenolic acid with antifungal activity against *Fusarium* species, is called ferulic acid [36]. Phenolic extracts from *Spirulina* species showed high antifungal activity against *Fusarium* fungi [37]. Here, co-inoculation of 150 µg/mL M20 extract with four *F. graminearum* strains separately, showed inhibitory effects on the mycelia growth in different ratios. We suggest that the antifungal activity of M20 extract against *Fusarium* species is due to its high flavonoid content which may bind to the cell wall and perform general defense system against plant pathogens [38].

To further study the effect of M20 extract on *F. graminearum* growth, we quantified the amount of *F. graminearum* DNA found in the total DNA extracted from wheat samples collected from the field conditions. We found that point inoculation of M20 extract with *F. graminearum* spore suspension inhibited the pathogen development. Similarly, Skadhauge et al. [39] demonstrated that flavonoid, dihydroquercetin inhibited hyphal penetration of *F. graminearum* and *F. culmorum* into the grain testa in barely. Moreover, selenium nanoparticles (SeNPs) synthesized by *Lactobacillus acidophilus* ML14 controlled *F. culmorum* and *F. graminearum* growth based on their powerful antioxidant and antifungal activities and hence, counteracting drought and heat stress in wheat plant [40]. Flavonoid, 5-hydroxy-7,4′-dimethoxyflavone which was extracted from *Combretum erythrophyllum* leaves using solvent acetone, inhibited the growth of many *Fusarium* species including *F. graminearum* with a MIC value of 0.63 mg/mL [41].

A key aspect in the selection of M20 extract against toxigenic *F. graminearum* is the evaluation of its ability to counteract mycotoxin production. In our experiments, we could show that DON amount in wheat samples inoculated with both Fg and M20 extract was significantly reduced to 73% compared to samples that were inoculated only with Fg. Furthermore, we could show that amount of *F. graminearum* DNA was highly correlated to DON accumulation which were consistent with previous findings [3,42,43]. These results indicate that M20 extract contributes to the suppression of FHB via the degradation of the DON. On the other hand, some studies concluded that the antioxidant activity of the phenolic/flavonoid compounds is related to the inhibition of the mycotoxin biosynthesis [32,44]. For example, quercetin had a significant decreasing effect on neosolaniol (NEO) and diacetoxyscirpenol (DAS) mycotoxins as reported by Schöneberg et al. [38] and gallic, caffeic, and *p*-coumaric acids reduced mycotoxin levels produced by the toxigenic species [37,45]. Plant extracts of cinnamon, clove, lemongrass, oregano, and palmarosa have reduced the accumulation of DON in *F. graminearum*-infected grains [46]. The biosynthesis of the deoxynivalenol consists of the cyclization of the sesquiterpene ring, which is catalyzed by the tricodiene synthase enzyme, followed by eight oxygenation and four esterification reactions. These sequence reactions lead to the formation of DON and its acetylated intermediates [47,48]. Inhibition of DON by phenolic compounds may be attributed to the repression of the metabolic route, which could require the expression of a carrier protein and a network of regulatory genes.

## 4. Conclusions

In this study, we characterize the behavior of methanolic extract of *Z. bungeanum* (M20 extract) as a biocontrol agent against *F. graminearum* as well as DON accumulation. Here the main flavonoids found in the extract (quercetin, epicatechin, kaempferol-3-O-rhamnoside and hyperoside) significantly inhibited the growth of four different *F. graminearum* species. The Russian isolate Fg15 was the most inhibited strain with a percent inhibition of 48.5%. Amount of fungal DNA measured by qPCR in wheat samples inoculated with mixture of Fg strains and 100 µg/mL M20 extract was significantly decreased compared to the wheat samples that received Fg only. In addition, these flavonoids have a major role in repressing DON production in the treated groups. Therefore, methanolic extract of *Z. bungeanum* can efficiently be used as a natural fungicide against plant pathogenic fungi to protect wheat crop loss, instead of using synthetic pesticides.

## 5. Materials and Methods

### 5.1. Chemicals, Reagents, and Zanthoxylum bungeanum Plant

Deoxynivalenol standard was purchased from Cayman chemical via Biomol (CAS Number: 51481-10-8). Potato dextrose agar (PDA) was purchased from OXOID, Basingstoke, UK) and prepared by suspending 39 g in 1L MQ water and sterilized using an autoclave (CertoClav, Leonding, Austria) at 121 °C and three bars for 20 min. Agarose, tris base, hydrochloric acid, chloroform, and proteinase K were purchased from fisher scientific, Helsinki, Finland. Dimethyl sulfoxide (DMSO), acetonitrile, ethylenediaminetetraacetic acid (EDTA), sodium dodecyl sulfate (SDS), beta-mercaptoethanol (β-ME), isoamyl alcohol, ethanol, and isopropanol are from Merck, France. Midori green advance is from nippongenetics, Duren, Germany. Sodium chloride is from VWR-chemicals, Leuven, Belgium. *Zanthoxylum bungeanum* plant (*Z. bungeanum*) was extracted and fractionated in our previous study [32]. Briefly, the plant pericarps were extracted with petroleum ether followed by methanol. Drying this methanol extract with rotavapor resulted in crude methanolic extract. This crude extract was fractionated by silica gel column chromatography to twelve sub-fractions using solvent mixtures from low to high polarity. One sub-fraction produced by elution with 80% ethyl acetate and 20% methanol (called M20 extract) was used in this study.

### 5.2. Pathogen Inoculum Production

We used two isolates from Northern Europe (south-western Finland) and two isolates from Southern Europe (southern Russia) (Table 1). All strains were isolated as single spores from wheat. The Finnish strains (59065 and 59068) were isolated during 2017; the Russian strains (58703 and 58772) were isolated during 2014 and 2015 respectively. The Finnish strains belong to 3ADON genotype; the Russian strains belong to 15ADON genotype. Fungal and chemo-typing identification were confirmed as explained by Yli-Mattila et al. [31]. For macroconidia production, seven PDA plates from each strain were grown at 25 °C and were exposed to UV light for 4 h per day. In addition, some strains were grown on SNA (Synthetischer Nährstoffarmer Agar) medium (1 g KH_2_PO_4_, 1 g KNO_3_, 0.5 g MgSO_4_, 0.5 g KCl, 0.2 g glucose, 0.2 g sucrose, and 20 g agar per liter) with a small piece of sterilized filter paper at 25 °C in order to induce conidia production. A spore suspension from each strain was prepared separately. Then, mixture of conidial suspension from the 4 Fg strains was prepared with sterile Milli-Q water. Final concentration 4 × 10^5^ conidia/mL was prepared by counting the macroconidia under microscope using the haemocytometer (Burker, JH1405-8, Hawksley, UK) and stored at 4 °C.

### 5.3. In Vitro Bioassay—Effect of M20 Extract on Mycelium Growth

Before applying M20 extract in the field, preliminary antifungal experiments were carried out in the laboratory. The antifungal activity of the M20 extract against four pathogenic *F. graminearum* strains were determined by the growth rate method [49]. In this method, 150 µg/mL of M20 extract was spread on PDA medium and PDA medium without any plant extract served as control. All the plates were inoculated in triplicate with 1 mm mycelium plug of each freshly produced fungal strain in the center of the plate and incubated in the dark at 25 °C. When the colonies of the blank control group covered the plate, the colony diameter of each plate was measured. The percent of growth inhibition (%) was calculated from this formula; (%) = (Dc − Dt)/Dc × 100; where Dc represents the colony diameter of the blank control group, and Dt represents the colony diameter of the treated group.

### 5.4. Treatments of Wheat Grains by Methanolic Extract of Zanthoxylum bungeanum in the Field

The field experiment was conducted at the Southwest Finland region (Marttila local place, 60.63050209 N, 22.98930788 E). A mixture of conidia from four single-spore *F. graminearum* (i.e., MFG 59065, MFG 59068, MFG 58703, and MFG 58772) isolates from wheat was used for the artificial inoculation method in the field experiment. This study included four different treatments; control received 20 µL DMSO, treatment I: mixture of conidia from four *F. graminearum* (Fg) isolates, treatment II: mixture of conidia from four Fg isolates in equal amounts followed by 100 µg/mL M20 extract, and treatment III: 100 µg/mL M20 extract only. Quantity of *F. graminearum* DNA was determined by qPCR based on the *TMFg12* gene. The amount of DON produced was monitored by HPLC. In addition, coefficient of determination between *Fusarium* DNA and mycotoxin level was calculated in the four studied groups. The wheat cultivar Sibelius was sown with Tume KL 2500 H SC (2.5 m wide) in the field on 4 May 2021. In July 2021, we had mid-flowering in the wheat field, plants were inoculated using point inoculation method in the fourth flower from below [50]. After adding the inoculating agent, the randomly selected wheat heads were covered with a plastic bag, which has been removed after 24 h. The plants were then kept for one month before harvesting. All wheat seeds were collected (1.5 g/one wheat head) and ground using a coffee mill (Krups KM75 Coffee Grinder) as described before [41] and stored in the refrigerator.

### 5.5. DNA Extraction and qPCR Analysis

DNA was extracted from 100 mg ground wheat samples using the GenElute™ Plant Genomic DNA Miniprep Kit (Sigma-Aldrich, Darmstadt, Germany) according to the manufacturer instructions. Fungal DNA from the standard *F. graminearum* isolate Fg13 was extracted from pure cultures using the manual DNA extraction protocol as described by [51] with minor modifications. Briefly, 10 mg of fungal mycelium was mixed with 300 µL of lysis buffer (50 mM Tris–HCl, pH 7.9; 50 mM EDTA, pH 8.0; 150 mM NaCl; 1% SDS; 0.5 M beta-mercaptoethanol; and 600 µg/mL Proteinase K) and incubated at 65 °C for 3 h. Then, 100 µL 5M NaCl and 400 µL chloroform: isoamyl alcohol solution (24:1) were added. The organic and aqueous phases were thoroughly mixed by inversion and incubated at room temperature for 15 min. The samples were centrifuged at 12,000 rpm for 2 min to pellet insoluble material. The upper aqueous phase was transferred into a new tube, incubated for 15 min at 4 °C, and centrifuged for 2 min to precipitate the proteins. Then, the aqueous phase was collected once more avoiding the pellet on the bottom of the tube. DNA was precipitated by addition of 0.6 volumes of 100% isopropanol and incubated at room temperature for 2 min. The DNA was pelleted by centrifugation at 12,000 rpm for 2 min, and the supernatant was removed from the tube. The pellet was washed with 400 µL of 70% ethanol and centrifuged for 5 min. The DNA pellet was dried in Eppendorf Concentrator Plus for 2 min and re-suspended in 25 µL of TE buffer (10 mM Tris–HCl, 1 mM EDTA, pH 8.0).

The concentration of the isolated DNA was measured by using a fluorescence-based Qubit fluorometer (Invitrogen, Carlsbad, CA, USA) according to the manufacturer recommendations. DNA concentrations were confirmed using agarose gel electrophoresis. Five microliters of extracted DNA was run on a 1% (*w/v*) agarose gel containing 0.1 μg/mL of Midori green. DNA was visualized using GeneGenius Bio Imaging System (Syngene, Cambridge, UK). All DNA samples were stored in Elution buffer (TE-buffer) supplied by the DNA extraction kit at −20 °C until further analyses.

Forward and reverse primers TMFg12f (5′ CTCCGGATATGTTGCGTCAA 3′) and TMFg12r (5′ CGAAGCATATCCAGATCATCCA 3′), and probe TMFg12p (5′ TGAGAATGTCTTGAGGCAATGCGAACTTT 3′) were designed using the primer express program Version 2.0 (Applied Biosystems) by [52]. TMFg12 probe was labelled at the 5′ends with 6-FAM (6-carboxy-fluorescein) and at the 3′end with TAMRA (5-carboxytetramethylrhodamine) for the quencher. Primers and probe were diluted on the same day of the experiment. Three replicates of each dilution (0.5, 0.05, 0.005, 0.0005, and 0.00005 ng) of the fungal DNA were prepared and used to construct the standard curve. Non-template control (NTC) was used by adding water-only. Reaction mixture for *F. graminearum* DNA or wheat DNA was prepared in final volume 25 µL containing 12.5 µL iQ^TM^ Supermix (purchased from Bio-rad, Watford, UK), 100 nM primers, 100 nM probe, and 1 µL DNA standard or sample. Amplification was performed on icycler iQ^TM^ 96-well PCR plates (Bio-Rad, Watford, UK), sealed with Optical Adhesive Covers (Bio-Rad, Watford, UK). TaqMan quantitative PCR was performed in an iQ^TM^5 Real-time PCR detection system (Bio-Rad, Watford, UK). The PCR program consisted of 3 min at 95 °C, followed by 40 cycles of 10 s at 95 °C and 30 s at 55 °C. Ct values were obtained by using iQ^TM^5 optical system software and exporting the amplification results into an Excel file. The amount of *F. graminearum* DNA was calculated from the Ct values and standard curve equation (Y = −3.449X + 28.661). From this equation and Ct values, X value (log quantity) was calculated. The quantity was calculated from Equation (1). Final *F. graminearum* DNA (pg/ng total DNA) amount was calculated by dividing the quantity by total DNA concentration from Qubit.

(1) $$Quantity=10^{Log_{x}}$$

### 5.6. Extraction and Evaluation of Deoxynivalenol Accumulated in Wheat Heads

DON was extracted by the method described by [3] with some modifications. Briefly, 0.5 g from each ground wheat sample was added to 5 mL solvent extraction buffer CH_3_CN:H_2_O (80:20 *v*/*v*). The samples were extracted using Infors CH-4103 Bottmingen shaker at room temperature and 180 rpm for 90 min. The supernatant was separated by centrifugation at 20 °C, 4500 rpm for 5 min, transferred to a new 15 mL falcon tube and diluted with the same amount of extraction solvent CH_3_CN:H_2_O (80:20 *v*/*v*). Then, 800 µL from this extract was filtered through 0.2 µm syringe filter (VWR, Radnor, Pennsylvania, North America) and stored in dark vials at −20 °C until HPLC analysis. DON standard (50 µg) was dissolved in 500 µL acetonitrile, filtered through 0.2 µm syringe filter, and stored at −20 °C. Samples and standard (10 µL) were injected to HPLC which was LiChroCART (Agilent Technologies, Waldbronn, Germany) and an Agilent 1100 series device consisted of absorption and fluorescence detectors (Agilent Technologies, Palo Alto, Santa Clara CA, USA) and C18 reversed-phase (LiChrospher 100, 125 × 4 mm, 5 µm) column. The mobile phase consisted of acetonitrile and water (15:85 (*v*/*v*)) at a flow rate of 250 µL/min. The column temperature was 30 °C. The HPLC system was equipped with a UV detector and fluorescence with 220 nm wavelength. DON concentrations were calculated according to the retention times and the areas of the corresponding peaks on the chromatogram using Analyt-FC (Agilent Technologies, Palo Alto, Santa Clara, CA, USA) collector.

### 5.7. Statistical Analysis

Statistical analysis was performed with Origin (OriginLab, Northampton, MA, USA). *Fusarium* abundance (expressed as *F. graminearum* DNA/wheat DNA), and DON level (expressed as mg/kg flour) were subjected to ANOVA followed by a Tukey HSD post hoc test for multiple comparison and groups are considered significant if *p* < 0.05. We analyzed the relationship between fungal DNA and DON data by coefficient of determination (r^2^). The qPCR data were transformed by multiplying with 1000 or 10 in order to obtain a more normal distribution with the toxin data.

## Figures and Tables

**Table 1:** Strain ID, isolation source, year of production and genotypes produced by the four strains used in this study.

Strain Number	Strain ID	Isolation Source	Plant	Year	Genotype
2	MFG 59065	Southern Western (Finland)	wheat	2017	3ADON
5	MFG 59068	Southern Western (Finland)	wheat	2017	3ADON
13	MFG 58703	Krasnodar krai (Russia)	wheat	2014	15ADON
15	MFG 58772	Stavropol krai (Russia)	wheat	2015	15ADON

## Data Availability

Not applicable.
